# Destigmatizing Carbohydrate with Food Labeling: The Use of Non-Mandatory Labelling to Highlight Quality Carbohydrate Foods

**DOI:** 10.3390/nu12061725

**Published:** 2020-06-09

**Authors:** Christopher P.F. Marinangeli, Scott V. Harding, Andrea J. Glenn, Laura Chiavaroli, Andreea Zurbau, David J.A. Jenkins, Cyril W.C. Kendall, Kevin B. Miller, John L. Sievenpiper

**Affiliations:** 1 Pulse Canada, 920-220 Portage Avenue, Winnipeg, MB R3C 0A5, Canada; 2 Department of Biochemistry, Faculty of Science, Memorial University of Newfoundland, St. John’s, NL A1C 5S7, Canada; sharding@mun.ca; 3 Department of Nutritional Sciences, University of Toronto, Toronto, ON M5B 1W8, Canada; andrea.glenn@utoronto.ca (A.J.G.); laura.chiavaroli@alumni.utoronto.ca (L.C.); andreea.zurbau@mail.utoronto.ca (A.Z.); David.jenkins@utoronto.ca (D.J.A.J.); cyril.kendall@utoronto.ca (C.W.C.K.); john.sievenpiper@utoronto.ca (J.L.S.); 4 Clinical Nutrition and Risk Factor Modification Centre, St. Michael’s Hospital, Toronto, ON M5B 1W8, Canada; 5 Li Ka Shing Knowledge Institute, St. Michael’s Hospital, Toronto, ON M5B 1W8, Canada; 6 Division of Endocrinology and Metabolism, St. Michael’s Hospital, Toronto, ON M5B 1W8, Canada; 7 College of Pharmacy and Nutrition, University of Saskatchewan, Saskatoon, SK S7N 5E5, Canada; 8 General Mills Inc., Global Scientific & Regulatory Affairs, Golden Valley, MN 55427-3870, USA; kevin.miller2@genmills.com

**Keywords:** quality carbohydrate, dietary fibre, whole grains, health claims, glycemic index

## Abstract

Dietary carbohydrates are components of healthy foods, but many carbohydrate foods have recently been stigmatized as primary causes of diet-related risk factors for chronic disease. There is an opportunity to enhance efforts within the food landscape to encourage the consumption of higher quality carbohydrate foods. The use of labelling is one strategy that permits consumers to identify healthy carbohydrate foods at the point-of-purchase. This review discusses the regulatory frameworks and examples of associated non-mandatory food labelling claims that are currently employed to highlight healthy carbohydrate foods to consumers. The existing labelling frameworks discussed here align with established measures of carbohydrate quality, such as 1. dietary fibre nutrient content claims and associated dietary fibre-based health claims; 2. the presence of whole carbohydrate foods and ingredients that are intact or reconstituted, such as whole grains; and 3. low glycemic index and glycemic response claims. Standards from Codex Alimentarius, and regulations from Australia and New Zealand, Canada, Europe, and the United States will be used to illustrate the means by which food labelling can be used by consumers to identify quality carbohydrate foods.

## 1. Introduction

“Quality carbohydrate” is a relatively new term that has been introduced as a means of discussing the contribution of carbohydrate foods to healthy diets. While not formally defined, individual and aggregated measures of carbohydrate quality have been discussed and applied within the literature, and have included one or more criteria, including total dietary fibre, whole versus refined grains, glycemic index (GI) or glycemic response, solid-to-liquid carbohydrate ratio, carbohydrate-to-fibre ratio, whole grain-to-total grain ratio, and sugar content [1,2,3,4,5].

Dietary carbohydrates are components of many healthy foods, including dairy, fruits and vegetables, legumes, seeds and nuts, and whole grains, yet carbohydrate foods are often stigmatized publicly as primary causes of diet-related risk factors for chronic disease. However, similar to dietary fat, the term carbohydrate encompasses various food components that, on their own and within foods, can have a spectrum of benefits on physiological function and health within dietary patterns. Fundamentally, the digestible carbohydrates obtained from foods are a source of energy for cells. Non-digestible carbohydrates, including dietary fibres and resistant starches promote stool regularity, lower circulating LDL-cholesterol, blunt postprandial glycemic responses, encourage mineral absorption in the large intestine, and impose positive effects on the human intestinal microbiome [6]. The fact that carbohydrate-rich foods are emphasized in national dietary guidelines and within dietary patterns shown to reduce cardiovascular and diabetes risk factors demonstrates their value in healthy diets [7,8,9].

Across jurisdictions, labelling tools exist to help consumers identify foods that align with healthy dietary patterns; this includes foods of higher carbohydrate quality. From a regulatory perspective, many jurisdictions permit the use of nutrient content claims to communicate the presence of nutrients or other healthy food components, including dietary fibre, in foods. Health claims that refer to a physiological function or a health benefit could be supported by the presence of a carbohydrate, such as specific types of dietary fibre. Other claims and labelling programs communicate the presence of intact or reconstituted foods and ingredients, such as whole grains, that contain carbohydrates and other nutrients, but also align with a jurisdiction’s nutritional policies. While polarizing, low glycemic index (GI) or glycemic response claims have also been permitted where it has been acknowledged that a lower peak rise in postprandial glucose levels is a physiological benefit to consumers and a nutritional strategy for managing blood glucose levels amongst people with diabetes.

This review provides an overview of the existing regulatory frameworks and examples of associated non-mandatory food labelling claims that are currently employed to highlight high-quality carbohydrate foods to consumers. The labelling frameworks discussed align with established measures of carbohydrate quality, such as 1. dietary fibre content claims and associated dietary fibre-based health claims; 2. the presence of whole carbohydrate foods and ingredients that are intact or reconstituted, such as whole grains; and 3. low GI and glycemic response claims. Standards from Codex Alimentarius, and regulatory frameworks from Australia and New Zealand, Canada, Europe, and the United States (the US) will be used to illustrate the means by which food labelling is used to identify quality carbohydrate foods to consumers. The benefits of expanding labelling regulations to further encourage consumption of higher quality carbohydrate foods will also be discussed.

## 2. Defining Quality Carbohydrate and Considering Consumer Perception

The term “carbohydrate quality” can be controversial and is open to interpretation, not only from a scientific perspective, but also from the perspective of the consumer. One common feature is that quality carbohydrate foods refer to those foods that support healthy dietary patterns. Carbohydrates contribute significantly to diets around the world [10]. Indeed, carbohydrate foods are ubiquitous in the food supply, found in many forms (processed and unprocessed) with various physiological and health benefits. Therefore, it is reasonable that carbohydrate quality would not be defined by a single attribute. Often, dietary guidelines have focused on sugar, starch, and dietary fibre to inform the consumption of quality carbohydrate foods [11]. Some of these attributes are often quantified on the nutrition declaration labels of pre-packaged foods. However, in addition to these qualities, there are opportunities to use non-mandatory labelling to highlight attributes that permit the identification of quality carbohydrate foods, that resonate with consumers. For example, emphasizing the presence of whole grains, legumes, and fruits and vegetables within multi-component food products can capture the presence of dietary fibre and complex starch, but also promote vitamin and mineral intakes, along with other plant components (e.g., polyphenols, etc.) with health benefits.

With multiple domains of carbohydrate quality, the next challenge is leveraging labelling tools that encourage consumers to choose higher quality carbohydrate foods over lower quality carbohydrate foods. Increased consumption of refined and rapidly digestible carbohydrates, where dietary fibre, micronutrients, and in some cases, proteins have been removed, has been linked to the development of cardiometabolic diseases and some cancers [4]. Studies demonstrate that diets containing higher levels of dietary fibre and intact carbohydrate foods, such as whole grains, are associated with lower mortality and risk of chronic disease [12,13]. However, it seems that, in some cases, consumers have often extrapolated information referring to refined carbohydrate and negative effects on health to all types of carbohydrate and carbohydrate foods. In a recent study in Canada, when consumers from three major metropolitan cities were asked to use word associations to convey their feelings toward carbohydrates, negative descriptions revolved around overeating, weight gain, risk, and feelings of guilt [14]. In the same study however, participants distinguished between “good” and “bad” carbohydrate foods, where the former was associated with fruits and vegetables, dietary fibre, whole grains, and slowly digestible carbohydrates [14]. These findings mirror a recent consumer survey by the International Food Information Council Foundation, where 23% of US adults believed carbohydrates cause weight gain, which was second to sugar at 27% [15]. Conversely, only 13% of participants believed fats caused weight gain. While these perceptions stigmatize carbohydrates, in the same survey, over 80% of participates identified dietary fibre and whole grains as healthy foods [15].

From the consumer data, there is an opportunity to enhance efforts within the food landscape to encourage higher consumption of quality carbohydrates. As outlined previously, various measures of quality carbohydrates have been applied to foods and diets in a research setting to quantify their characterization as quality carbohydrate foods. While all measures of quality carbohydrates used academically may not be suitable for labelling initiatives, there are broad domains of carbohydrate quality that can and are already used in the marketplace. In 2017, a workshop hosted by the International Life Science Institute North America put forth vision statements that identified three domains of quality carbohydrate foods: 1. a source of dietary fibre; 2. whole food credentials; and 3. low GI or glycemic response. These three domains are closely related to those used in a systematic review and meta-analyses of carbohydrate quality on chronic disease by Reynolds et al. [16]. In addition to dietary fibre and the GI, rather than a broad evaluation of whole foods, whole grain foods were specifically reviewed. Across regions, regulatory frameworks and dietary guidelines already permit the use consumer-facing non-mandatory labelling tools that align with these domains of quality carbohydrates.

The use of labelling is one strategy that permits consumers to easily identify healthy foods at the point-of-purchase. The following sections of this review will discuss and summarize regulations and provide non-mandatory labelling examples that have been used across jurisdictions that have been leveraged to facilitate higher consumption of quality carbohydrates. While some labelling initiatives must follow specific compositional criteria for claims, other labelling initiatives communicate the presence or an attribute of the food. Fundamental to all labelling initiatives, it is imperative that the information communicated to the consumer is not misleading.

## 3. Labelling Foods for Carbohydrate Quality

### 3.1. Dietary Fibre

#### 3.1.1. Direct Dietary Fibre Claims: Fibre Nutrient Content Claims

The presence of dietary fibre is a commonly identified measure of carbohydrate quality. Although dietary fibre is not a nutrient per se, it is considered to be a beneficial component of dietary patterns. Across regions, Australia and New Zealand, Canada, Europe, and the US have set specific regulatory targets for dietary fibre consumption as well as dietary fibre nutrient content claims (Table 1). In the US and Canada, although a recommended daily allowance (RDA) has not been established, an adequate intake of 14 g fibre/1000 kcal is recommended and is based on reduced risk for coronary heart disease [17,18,19]. In Australia and New Zealand, adequate intakes for dietary fibre of 14–30 g/day were derived from median intakes of fibre in populations where issues with laxation did not occur [20]. Similarly, dietary fibre recommendations in Europe are based on effects on laxation with 25 g fibre/day recommended for adults (2–3 g fibre/MJ), and 2 g fibre/MJ for children ≥1 year of age [21]. Given that daily dietary fibre recommendations are relative to energy intake, recommendations can differ between life stages. Note that Codex Alimentarius implements food standards that consider the input from membership countries with different food landscapes, and dietary recommendations for dietary fibre have been left to individual countries [22].

Despite different dietary fibre recommendations across regions, recommendations are based on the observation that dietary fibre can improve physiological function or prevent chronic disease and supports the value of identifying high fibre foods as nutrient content claims. While the criteria differ, nutrient content claims provide a fundamental platform for communicating that foods are a source of quality carbohydrates. However, confusion can arise because of differences in the definition of dietary fibre across regions. As outlined in Table 2, definitions of dietary fibre commonly include indigestible carbohydrates from plants. With the exception of Codex, a degree of polymerization of monomeric units of ≥3 is common among Australia and New Zealand, Canada, Europe, and the US. For extracted and/or novel dietary fibres (including synthetic fibre), a physiological benefit must be demonstrated before the carbohydrate can be considered a dietary fibre. Laxation, cholesterol-lowering, and decreased postprandial glucose and insulin responses are common physiological benefits between countries. However, the US has an expanded list that includes mineral absorption and effects on energy intake from food consumption. Canada and Europe have also included microbial fermentation in the large intestine. However, the directive from the European Commission that outlined the accepted physiological benefits for novel dietary fibres was repealed [28] and replaced by regulation 1169/2011 [29]. It is assumed that the physiological benefits outlined in the previous directive remain as acceptable. Canada explicitly indicates that other benefits not outlined in the dietary fibre policy could also be accepted for novel fibre sources [30].

The common ability to claim that foods are a source dietary fibre is a straightforward opportunity for consumers to identify food sources of quality carbohydrates. For industry, studies to substantiate accepted physiological benefits of extracted, novel, or synthetic dietary fibres can be challenging to demonstrate in healthy populations, but are minimally invasive. However, given that the requirements for dietary fibre claims can differ across jurisdictions, similar foods may not always have the ability to leverage “source of fibre” claims in different countries. Nevertheless, consumers and industry have access to many fibre-containing unprocessed and processed foods, and fibre ingredients, respectively, that can be leveraged as an attribute of quality carbohydrates.

#### 3.1.2. Indirect Dietary Fibre Claims: Function and Disease Risk Claims

Health claims that communicate a functional or health benefit from the presence of a specific type of dietary fibre could also be used to increase the consumption of quality carbohydrates. Functional-type health claims (general level health claims in Australia and New Zealand) refer to a physiological benefit from the food. Therapeutic or disease risk reduction health claims (high-level health claims in Australia and New Zealand) refer to effects of a food or ingredient on chronic disease risk factors such as cholesterol and blood pressure lowering, or disease prevention. Recall that physiological and health benefits can be used to characterize novel carbohydrate ingredients as dietary fibres (see Section 3.1.1.). However, the criteria for leveraging physiological and health benefits as a standalone claim on foods from the inclusion of dietary fibre can require higher standards of evidence, and can differ between regions.

Table 3 provides examples of physiological function claims that have been identified by regulatory agencies across regions that are based on the presence of dietary fibres. Laxation claims are common. In Australia and New Zealand, all dietary fibres can claim an effect on laxation if the levels of fibre within a food meet the general conditions for a fibre nutrient content claim (Table 1: 2 g/serving). This is reasonable given that dietary fibre recommendations are based on laxation. This is similar to Europe where claims related to increasing fecal bulk, decreased transit time, or normal bowel function can be used if the level of fibre in the food qualifies for a “high in fibre” claim. In Canada, the Canadian Food Inspection Agency (CFIA) has indicated that function claims referring to the effect of wheat bran and psyllium on laxation are permitted. Claims referring to a reduced postprandial glycemic response, maintenance of normal cholesterol levels, and contribution to weight loss in the context of a calorie-restricted diet are also considered to be function-type health claims in Europe and have been approved for a variety of dietary fibres.

In Europe, all claims regardless of their scope must be reviewed by the European Food Safety Authority and subsequently added to EU regulation 432/2012 [36]. In the US, structure/function-type health claims do not undergo pre-approval, and thus a list of function claims is not provided within the US Code of Federal Regulations but does not preclude their use on food labels [39]. In some regards, the US is similar to Australia and New Zealand, and Canada, where function-type claims do not require regulatory approval. However, regulatory agencies in these regions will review function-type claims if requested, and subsequently publish their assessment and approval. An example of this has been demonstrated in Canada, where a proprietary combination of viscous fibres characterized as a “polysaccharide complex (glucomannan, xanthan gum, sodium alginate)” was reviewed by Health Canada and accepted as an ingredient that can lower the postprandial glycemic response [35]. In Australia and New Zealand, if a review is not requested prior to utilization, the Chief Executive Officer of Food Standards Australia New Zealand must be notified of the claim [33]. In all three regions, function-type claims used by industry that have not undergone review are required to have adequately substantiated the claim internally and could be asked by regulators to present a claim dossier.

In addition to fibre claims that disseminate an effect on physiological function, various fibres have been reviewed and approved for claims that communicate their ability to decrease cardiometabolic disease risk factors (Table 4). Across the regions included in this review, claims that promote the cholesterol-lowering efficacy of beta-glucan from oats and barely are permitted. For Australia and New Zealand, and Europe, a minimum of 1 g/serving beta-glucan is required to make a cholesterol-lowering claim [23,40,41]. In Canada, at least 0.75 g beta-glucan from oat and 1.0 g beta-glucan from barley per reference amount and serving of the stated size of a food are required [42,43]. In the US, the minimum level of beta-glucan for a lower risk of coronary heart disease claim is 0.75 g per reference amount customarily consumed (RACC) [44]. For all regions summarized, labelling must also communicate a contextual statement that 3 g/day beta-glucan is required. In Europe, it is important to highlight the distinction between the effect of fibres on maintaining cholesterol levels as a function claim (Table 3) and cholesterol-lowering as a risk reduction claim (Table 4). Canada has approved cholesterol-lowering claims for soluble psyllium fibre at 1.75 g/reference amount (and serving of stated size) and 7 g/day [45]. A similar claim referring to a lower risk of coronary heart disease risk is permitted in the US for soluble psyllium fibre at 1.7 g/RACC (US) (and 7 g psyllium fibre/day) [44]. The cholesterol-lowering efficacy of a proprietary polysaccharide complex has also been approved as a cholesterol-lowering ingredient in Canada [46]. The US has authorized health claims for high-fibre grains, fruits, and vegetables for their effects on decreasing the risk of coronary heart disease and cancer, and is based on those grains, fruits, and vegetables that contain at least 0.6 g soluble fibre per RACC [47] and is at least a “good source of fibre” [48] (Table 2), respectively. Regulations indicate that numerous fibre ingredients have been approved for claims relating to physiological benefits and reduced risk for cardiometabolic disease, which are based on the presence of fibre as a quality carbohydrate source.

### 3.2. Emphasis on Whole Foods

Whole foods, such as whole grains, or their presence in multicomponent and manufactured foods, can resonate with consumers as healthier food options. When intact foods or all of their components are consumed, it can facilitate the consumption of quality carbohydrates, as well as vitamins, minerals, and other possible bioactives that are often removed when ingredients are refined.

Randomized clinical trials and prospective cohorts studies have demonstrated that higher consumption of whole carbohydrate foods, such as low-fat dairy, legumes, whole grains, nuts, fruits, and vegetables, have been shown to decrease disease risk factors and/or are associated with reduced disease incidence [13,49,50,51,52,53,54,55,56,57,58,59,60,61,62,63,64]. Options for labelling that a multicomponent food contains whole food ingredients that are intact or reconstituted to the proportions of their native form are often permitted. This has been demonstrated with labelling programs that highlight whole grains.

The use of food labelling to identify the presence of a broad category of quality carbohydrates within foods, like whole grains, that align with consumer perceptions of a healthy dietary pattern and dietary guidelines could be an effective labelling tool for the consumer. Whole grain cereals and pseudocereals can be consumed as intact cereals, as in the case of brown rice and whole oats (groats), or used as ingredients in multi-component foods. The Cereals & Grains Association (formally the American Association of Cereal Chemists) has defined whole grains as cereals and pseudocereals that “consist of the intact, ground, cracked or flaked caryopsis, whose principal anatomical components—the starchy endosperm, germ and bran—are present in the same relative proportions as they exist in the intact caryopsis [65].” Australia and New Zealand have formally adopted a similar definition of whole grains in the Food Standards Code [66]. Similar definitions of whole grains have been provided by Health Canada and the US as statements or proposed guidance, respectively [67,68]. In Europe, a legal definition of whole grains for use in human food has not been established with different definitions of whole grains being used across countries [69]. The European Food Safety Authority has referenced Cereals & Grains Association’s definition in an opinion for health claims related to whole grains [70]. Despite established definitions, without some level of dietary knowledge, it could be difficult for some consumers to identify foods that are indeed whole grains or contribute a meaningful amount of whole grains expected to convey some health benefit. Whole grains are emphasized in most dietary guidelines in Europe, as well as Canada, the US [71], Australia [9], and New Zealand [72], with evidence demonstrating dose-dependent relationships between higher whole grain consumption and reduced risk of all-cause mortality, coronary heart disease incidence, type 2 diabetes, and colorectal cancer [12,13,16].

**Figure 1:**
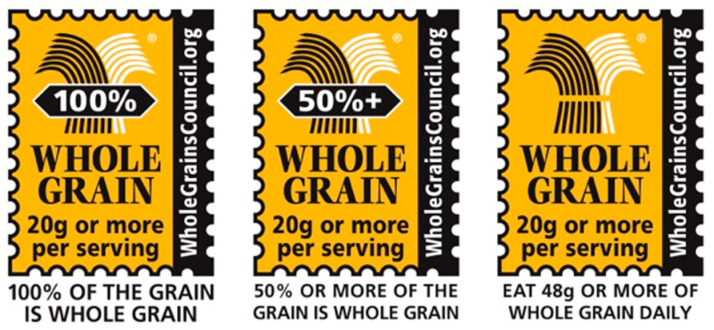
Examples of the Oldways Whole Grains Council’s Whole Grains Stamp that assists consumers to identify foods that contain significant levels of whole grains [73]. Reproduced with permission from the Oldways Whole Grains Council.

Messaging that identifies wholes grains within the marketplace, such as oats, could help increase consumption, regardless of whether the consumer is knowledgeable about quality carbohydrate foods. Labelling statements or symbols that indicate that these foods contain a significant level of whole grains can also facilitate increased consumption. As an example, The Oldways Whole Grains Council has developed and implemented a Whole Grain Stamp labelling program that communicates the presence of whole grains in food products in 62 countries, including Canada and the US (Figure 1) [73]. Utilization of the front-of-pack labelling symbol is contingent on a minimum of 8 g/serving of whole grains, which is one-half of the US Department of Agriculture’s defined serving of whole grains (16 g) [8]. Similarly, Australia’s Grains & Legumes Nutrition Council implemented a voluntary code of practice for claiming that foods are a source of whole grains. The code permits the use of whole grain claims on foods to indicate they contain ≥8 g/serving (“contains whole grain”), ≥16 g/serving (“high level in whole grain”), or ≥24 g/serving (“very high in whole grain”) [74]. Additionally, all general and health claims in Australia and New Zealand must comply with the Nutrient Profiling Scoring Criterion (NPSC) [33] outlined in Schedule 4 of the Food Standards Code [23]. A systematic audit of foods in major retail outlets in Sydney showed that utilization of whole grain content claims increased by 71% across food categories evaluated between 2013 and 2019 [75]. Although whole grain labelling has been discussed in detail, similar programs that emphasize the nutritional contribution of other whole quality carbohydrate foods could also be developed. It is also worth noting that front-of-pack labelling symbols cannot be used in a manner that interferes or detracts from mandatory nutrition labelling [29,76,77,78].

Although similar, claims that emphasize the presence of a whole food by using “made with” or “contains” claims do not necessarily have the same utility as front-of-pack symbols or claims that are supported by nutritional and dietary guidance. Consumers may not understand that the former is often solely based on the presence of the ingredient and is not necessarily founded on the ingredient’s contribution to a healthy dietary pattern, and, in the context of this review, quality carbohydrates. For example, Canada’s “Safe Food for Canadians” regulations do not permit the use of words or symbols that falsely communicate the presence of an ingredient [79]. The CFIA’s corresponding policy on highlighted ingredients indicates that “it is misleading to over-emphasize the importance, presence or absence of an ingredient or substance in a food because of its desirable or undesirable qualities, or for any other reason [80].” While one could extrapolate this to the presence of an ingredient, such as a quality carbohydrate food or ingredient, ambiguous claims or symbols may not provide sufficient information to the consumer that the claim is referring to nutritional or dietary criteria. For example, an ambiguous claim highlighting the presence of the ingredient could refer to attributes other than nutrition, such as flavour, texture, or the absence of artificial ingredients.

The US and Australia and New Zealand do not have regulations and policies that qualify the use of claims that communicate the presence of particular ingredients. An analysis of fruit and vegetable “presence,” “proportion,” or “serving” claims in Australia demonstrated that 31%, 52%, and 8% did not meet the cut off from the NPSC, respectively [81]. In some cases, without a reference level, whole food claims could be challenging and, if not implemented correctly, could trigger enforcement from regulatory agencies.

### 3.3. The Glycemic Index and Glycemic Response

The GI is a measure of the postprandial glycemic response of a carbohydrate food relative to an equal carbohydrate portion of a reference food as liquid glucose or white bread. Postprandial glycemic responses are measured directly on a glucose scale or converted to the glucose scale when bread is used as the reference food [82]. The test food and reference food are consumed in servings that contain 50 g of available carbohydrates. When levels of carbohydrates are low in the test food, 25 g available carbohydrates is used for both the test and reference food [82]. Given that the GI is determined by using a standardized reference (glucose or bread), foods can be characterized as having a low (<55), medium (56–69), or high (≥70) GI (based on a glucose scale) [82,83]. The GI is only applicable to foods with physiologically relevant levels of available carbohydrates per serving [84]. Many foods with significant levels of carbohydrates that also have a low GI are acknowledged in dietary guidelines across regions and include specific whole grains, legumes, nuts, dairy, temperate climate fruits, and a variety of vegetables [85].

From a scientific perspective, the GI has been successfully used as part of a diet-based approach to manage blood glucose levels in individuals with diabetes [86,87,88,89,90,91]. Guidelines for the management of diabetes in Canada, Australia, Europe, the UK, and the US acknowledge that low GI dietary patterns can be used to assist with blood glucose management [92,93,94,95]. In Canada, low glycemic index diets have also been acknowledged as a strategy for the prevention and management for cardiovascular disease [96].

From a regulatory perspective, the GI is the most contentious labelling strategy for identifying quality carbohydrate foods. In a recent systemic review and meta-analysis of prospective cohorts, Reynolds et al. [16] concluded that, compared with dietary fibre and whole grains, the GI might not be as useful a measure of carbohydrate quality for the prevention of chronic disease. Conversely, subsequent dose–response meta-analyses of prospective cohorts showed that the risk of coronary heart disease and type 2 diabetes increased by 24% and 27% per 10 unit increase in GI, respectively [97,98]. Food Standards Australia New Zealand permits “low,” “medium,” and “high” GI claims. However, historically, GI claims have not been permitted in Canada and Europe [99,100]. To our knowledge, there is no regulation in the US that would discourage GI labelling on food.

The rationale for not permitting the use of GI labelling are multifaceted and include the following: perceived challenges with the precision and accuracy of the methods used to measure the GI [101], risk of low-GI foods misaligning with regional healthy eating policies [99], and poor characterization of low-GI foods [100]. Uncertainties around the precision and accuracy of GI values have largely been addressed in the scientific literature [101]. The International Organization for Standardization (ISO) has published an official method for determining the GI of a food [84]. A recent study demonstrated that the ISO method generated accurate GI values with an interlaboratory standard deviation of 5.1% and a coefficient of variation of 8.1% [102]. Results also showed that the ISO GI method was sufficiently precise to distinguish between low- and high-GI foods with 97–99% probability [103]. From a labelling perspective, it is valid that published tables on GI values can demonstrate variability between similar products [85]. However, as with any labelling framework, it is the responsibility of the industry stakeholder using the claim to ensure that the GI of a specific product is assessed using a validated method and confirmed to have a low GI, and not extrapolated from other data sources.

**Figure 2:**
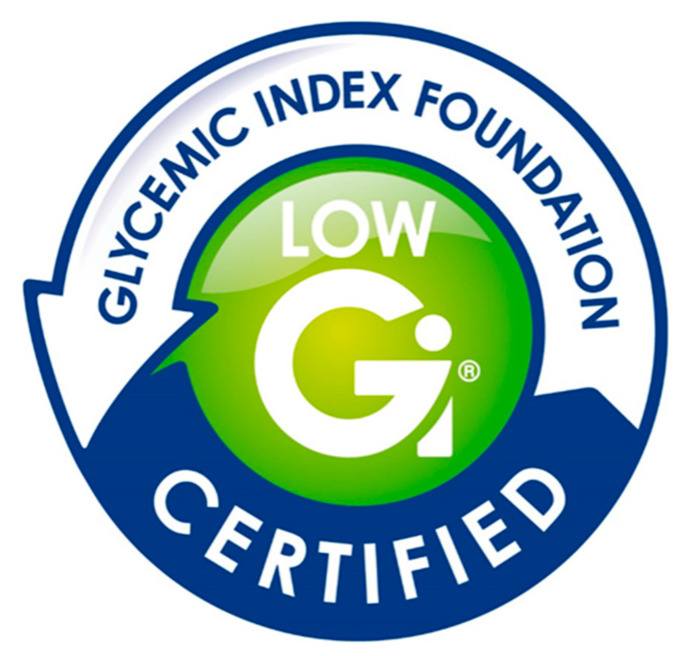
The Glycemic Index Foundation’s low glycemic symbol used by consumers to identify that foods have a low GI [104]. Reproduced with permission from the Glycemic Index Foundation.

The GI is an attribute of the food. Thus, it is fair that some low-GI foods may or may not align with national dietary policies. Health Canada has outlined concerns that snack-type foods, such as ice cream, and naturally or artificially sweetened beverages could be classified as low-GI foods and mislead consumers to perceiving these foods as healthy and encourage consumption [99]. However, mechanisms can be implemented to mitigate this risk. For example, in Australia and New Zealand, health claims, including GI claims, can only be made if food products meet specific nutritional criteria quantified by the NPSC [23]. The Glycemic Index Foundation (GIF) is an Australia-based non-profit organization supported by the University of Sydney, and Diabetes New South Wales and the Australian Capital Territory, that provides the food industry with a front-of-pack GI symbol program to permit consumers to quickly identify low-GI foods in the marketplace (Figure 2). In addition to regulatory requirements, the GIF has additional nutritional and testing requirements before the symbol program can be used on food products [103]:

1. The food must contain ≥7.5 g carbohydrate/serving, or be ≥80% carbohydrate (served in multiple units of small servings sizes as part of one meal or snack) [104];
2. The GI of the food is measured using the ISO method [104];
3. The nutritional profile of foods meet category-specific criteria for energy, saturated fat, sodium, and dietary fibre, specified by the GIF [104];
4. Adhere to the GIF’s glycemic index testing policy [105].

The GIF symbol program focuses on the identification of “low-GI” foods (GI ≤ 55) and negates the need to linking the symbol to a GI number, which could cause confusion amongst consumers. It is generally accepted that “low-GI” foods have a GI value ≤ 55, which has been used as the cut-off for demonstrating beneficial effects on blood sugar management and reduced cardiometabolic risk. Data from the Australian National Nutrition Survey demonstrated that the GI and glycemic load of diets had decreased by 5% and 12% respectively, from 1995 to 2012 [106]. Combining the GI with nutrition profiling ensures that potential benefits of decreasing postprandial glycemic responses from carbohydrate foods are not counteracted by dietary factors associated with unhealthy dietary patterns.

Regulations in Australia and New Zealand also permit “medium GI” and “high GI” claims on food. However, the latter two claims have little, if any utility for the consumer for identifying quality carbohydrate foods. Again, the benefits of the GI as a tool to facilitate healthy carbohydrate choices are supported by patterns that incorporate foods with a “low GI” designation. Thus, adopting GI as a labeling strategy is only supported by foods with a GI ≤ 55. It is also reasonable that when a food is reformulated, it is retested to ensure that it qualifies for a low GI designation.

Although Canada and Europe do not permit labelling to identify low-GI foods, both jurisdictions have been receptive to the use of postprandial glycemic response claims, which itself is also considered a function-type health claim (Table 3). Similar to the GI, the postprandial glycemic response is determined by measuring the incremental area under the blood glucose curve of the test food. However, rather than indexing against a standardized control, in theory, any food can be used as the reference food. Considered to be a function-type health claim, in 2013, Health Canada published a draft guidance document for postprandial glycemic response claims, where reference foods were suggested to be similar to the test foods [107]. It was also indicated that the postprandial glycemic response should be at least 20% lower than the reference food without a disproportionate rise in insulin levels to make the claim [107]. Few stakeholders in Canada have applied glycemic response labelling to foods as the approach can be limiting to stakeholders. Furthermore, given that the proposed claim is relative to a specific food, the incorporation of “low glycemic response claims” and its efficacy for blood glucose management through the adoption of dietary patterns is arbitrary. While a review for function-type health claims is not required, Health Canada has reviewed and approved a low glycemic response claim for a proprietary polysaccharide complex that contains various viscous dietary fibres (glucomannan, xanthan gum, and sodium alginate) [35]. Reference to the control food has not been identified in the claim statement [35] and is a departure from Health Canada’s draft guidance [107]. In Europe, similar to the GI, The European Food Safety Authority (EFSA) has published the opinion that carbohydrate foods that induce a low glycemic response are insufficiently characterized [21]. Nevertheless, since 2010, numerous dietary fibres in Europe that have been appropriately characterized have been authorized to facilitate “a reduction in blood glucose rise after a meal” (Table 2).

An overarching challenge with glycemic response claims is that they are indiscriminate. Labelling and advertising that a food reduces the postprandial glycemic response is only relative to the reference food used. While a significant decrease in the glycemic response might be observed with test food compared with the reference food, the response may not be particularly useful for individuals with diabetes or impaired glucose tolerance. Thus, the utilization of the postprandial glycemic response in the context of a dietary pattern, which would be required to demonstrate meaningful benefits of blood glucose management and decreased cardiometabolic risk over time, can be difficult for the consumer to implement and quantify. On the other hand, low-GI foods, with values ≤55, are characterized by comparing a test food to a standardized reference of glucose (or bread). This enables foods to be definitively labelled as “low-GI foods” and incorporated into dietary patterns that can be adopted and, over time, result in a predictable outcome, which corresponds with the purpose of a claim and quality carbohydrates.

## 4. Discussion

There are multiple opportunities to use labelling to promote the consumption of quality carbohydrates. Given that carbohydrate is a macronutrient, high-quality carbohydrate foods can encompass various characteristics. For the most part, source of dietary fibre, dietary fibre-related health claims, whole carbohydrate foods, including whole grains, and low GI and response claims have been implemented internationally into non-mandatory labelling strategies to assist consumers with choosing carbohydrate foods that align with nutrient-dense dietary patterns and/or prevent non-communicable diseases. However, as demonstrated in this review, although the parameters of carbohydrate quality are similar, regulatory frameworks corresponding to quality carbohydrate criteria can differ between regions.

Just as there is no “one-size-fits-all” healthy dietary pattern, this review demonstrates that there are multiple attributes that can be used to highlight carbohydrate quality in foods to the consumer. The laws that underpin regulations and policies that permit the characteristics of foods to be communicated to the consumer are predicated on the fact that food labels and claims cannot be misleading [29,108,109,110,111,112]. Labelling for “source of fibre,” fibre-derived health claims, and programs that highlight the presence of whole food credentials are straightforward with similar claims made across jurisdictions, whereas labelling with regard to the GI and glycemic response continues to be debated and varies across regions.

The effects of carbohydrate foods on postprandial glycemia are the most contentious measure of carbohydrate quality. The ongoing polarized debate around “low GI” labelling frameworks is an example of the disconnect between developments in nutrition science and labelling regulations. Jurisdictions have acknowledged the value of managing postprandial glycemia, which, over time, can assist with decreasing the risk of vascular complications linked to diabetes [113]. Australia and New Zealand permit claims that identify foods as “low GI,” which has been successfully leveraged and implemented by the GIF. Canada and Europe have been transparent by presenting their rationale for refuting labelling claims that identify low-GI foods or permitting foods that facilitate a lower glycemic response. Although the GI is an attribute of the food that is not presented in the same manner as glycemic response, scientific validity has been presented regarding its benefits when used to help facilitate blood glucose management. This review has highlighted that various metrics of quality carbohydrates exist, and will resonate differently for consumers depending on their food values and needs. Canada’s position on GI labelling contradicts Canadian expert opinions for the management of diabetes and cardiovascular disease risk since guidelines recommend low-GI dietary patterns [92,96]. A recent study demonstrated that Canadians would be receptive to GI labelling as a tool to destigmatize carbohydrates and assist with choosing healthy carbohydrate foods [14]. Similar to Australia and New Zealand, when used alongside nutrient profiling, GI labelling can be an efficacious strategy for implementing dietary patterns with higher carbohydrate quality.

Sugar content in foods has not been included as a domain used to identify quality carbohydrate foods. Given that the levels of sugar in specific foods have been raised as a nutritional concern for its effects on cardiometabolic risk, characterizing foods with a high level of sugar as foods of lower quality carbohydrate could be considered. Across regions, policies, including mandatory front-of-pack labelling initiatives, have been used to help consumers choose foods with lower levels of sugar [114]. However, while sugar is a carbohydrate, the absence of sugar is not necessarily a proxy for the carbohydrate quality of foods. Although foods with higher levels of added sugars are linked to cardiometabolic risk, risk is not ubiquitous across all food types. High consumption of added sugars in sugar-sweetened beverages (SSBs) have been consistently shown to be associated with increased risk for diabetes and cardiovascular disease [12,13,115,116]. Conversely, total sugar consumption or intrinsic sugars in solid and liquid foods have not demonstrated the same associations [117,118,119]. A global review on the effects of dietary factors on the global burden of disease identified higher consumption of SSBs as a significant contributor to disability-adjusted life years (DALYs) and deaths from cardiovascular disease (DALYs: 2.8 million; deaths: 117 thousand) and type 2 diabetes (DALYs: 1.6 million; deaths: 21 thousand) [120]. Other sugar-containing foods were not identified. Comparatively, low consumption of other quality carbohydrate foods, such as fruit (DALYs: 65 million; deaths: 2.4 million), vegetables (DALY: 34 million; deaths: 1.5 million), whole grains (DALYs: 82 million; deaths, 3.1 million), nuts and seeds (DALYs: 50 million; deaths: 2.1 million), legumes (DALYs: 11 million; deaths: 535 thousand), milk (DALYs: 2.7 million; deaths: 126 thousand), and dietary fibre (DALYs: 20 million; deaths: 873 thousand), was ranked higher than increased SSBs consumption as dietary factors that prevent non-communicable diseases [120]. The purpose of mandatory front-of-pack labelling for added sugars in foods, is in part, to prevent high intakes of added sugars that could displace healthy food options from the diet and increase the risk of cardiometabolic disease [121]. However, in the context of labelling for quality carbohydrate foods, sugar on its own, may not be as useful as other domains outlined in this review [122].

It is important to acknowledge that, from an academic perspective, measurements of carbohydrate quality in dietary patterns can be more comprehensive than parameters used to inform consumer food choices. The SUN cohort used a comprehensive approach that combined attributes of carbohydrate quality outlined in this review (dietary fibre and the GI), as well as other indicators (whole grains-to-total grains ratios, solid-to-total carbohydrate ratio) to determine associations with obesity, cardiovascular disease incidence, and micronutrient intake adequacy [1,123,124]. It is undetermined if these approaches are useful as a labelling strategy. Mozaffarian et al. [5] compared various strategies for identifying whole grain foods in grocery stores, which included the Whole Grain Stamp cited in this review. Results demonstrated that using a ≤10:1 ratio of carbohydrate-to-fibre was the most effective at identifying foods with higher levels of dietary fibre, and lower levels of sodium, and sugar. While promising, this approach could require a regulatory assessment. The successful implementation of any labelling strategy by industry would require consumer education and industry support. Labelling concepts that are overly complex may hinder their adoption and usefulness in the marketplace.

Labelling that highlights the quality carbohydrates of a food is not mandatory. It is ultimately up to industry stakeholders to decide on the labelling messages that best align with the foods provided to their consumers, and then for consumers to choose attributes of carbohydrate quality that align with their dietary needs and values. The attributes of a food and corresponding labelling strategy will differ depending on the targeted consumer, and their preferences. Having multiple labelling tools at the industry’s disposal across multiple domains of carbohydrate quality can assist with the widespread consumption of quality carbohydrate foods. The scientific community ensures that parameters used to characterize quality carbohydrates, or other attributes, are scientifically valid and applied within a regulatory framework that is not misleading to the consumer. The food environment must enable scientifically valid labelling tools to be accessible to industry to facilitate innovation across multiple parameters of carbohydrate quality.

In the regions discussed in this review, mandatory nutritional information is required on most prepackaged food products. Fresh foods or some single ingredient foods are exempt from nutrition labelling [18,29,125,126]. In Canada and the US, the dietary fibre content of a food per serving is a mandatory component of nutrient declaration panels [18,125], while it is optional in Australia and New Zealand, and Europe, unless a food presents a dietary fibre nutrient content claim [29,126]. While claims and front-of-pack labelling can be useful for quickly identifying the carbohydrate quality of foods, fundamental nutritional literacy and habitual use of mandatory nutrition information on products could help consumers better evaluate foods for their nutrition value, including quality carbohydrates, and make better selections, and thus enhance the nutritional quality of their diets. Buyuktuncer et al. [127] demonstrated that students that had consistently used nutrition facts tables on food products had higher composite Healthy Eating Index-2005 scores, and higher intakes of total fruit, whole fruit, total vegetables, whole grains, and milk. Consistent users also had higher scores associated with lower intakes of added sugars [127].

## 5. Conclusions

Over the last decade, carbohydrate foods have been increasingly stigmatized by consumers. While some types of carbohydrate (i.e., sugar) may not confer a nutritional or health benefit, regulatory agencies and dietary guidelines recognize carbohydrate-rich foods and specific carbohydrate fractions, such as dietary fibre, to be part of healthy dietary patterns. High-quality carbohydrate foods can be identified as those that are high in dietary fibre, contain meaningful levels of whole carbohydrate foods and ingredients that are intact or reconstituted, such as whole grains, or have a low GI or glycemic response. Regulatory agencies around the world permit the use of non-mandatory labelling tools to promote quality carbohydrate foods to consumers across these domains of carbohydrate quality. While not exhaustive, this review provided examples of quality carbohydrate labelling that has been leveraged across Australia, New Zealand, Canada, Europe, and the US. This review does not promote one labelling strategy over another and acknowledges that different facets of carbohydrate quality will resonate differently between consumers. However, as nutrition science continues to evolve, it is crucial that government agencies are equipped to adapt to developments in nutrition science to ensure regulatory frameworks enable the use of labelling to relay messages that destigmatize carbohydrates and steer consumers to healthy quality carbohydrate choices.

## Figures and Tables

**Table 1:** Summary of dietary fibre recommendations and criteria for nutrient content claims for dietary fibre from Codex and in Australia and New Zealand, Canada, Europe, and the US.

	Codex Alimentarius Standards [22]	Australia and New Zealand [20,23]	Canada [17,19]	Europe [21,24]	United States [17,25]
Dietary fibre Recommendation	Recommendation to be determined at the national level	14–30 g/day (based on median intakes to prevent laxation)	14 g/1000 kcal	2–3 g/MJ (239 kcal)	14 g/1000 kcal
Basis for Dietary fibre Recommendation	N/A	↑ Laxation	↓ Coronary heart disease risk	↑ Laxation	↓ Coronary heart disease risk
Dietary fibre Nutrient Content Claims	**Source** ■ 3 g dietary fibre per 100 g; or ■ 1.5 g dietary fibre per 100 kcal; or ■ 10% of the DRV per serving **High Source** ■ 6 g dietary fibre per 100 g; or ■ 3 g dietary fibre per 100 kcal; or ■ 20% of the DRV per serving	**General Claim** ■ A serving of the food contains at least 2 g of dietary fibre. **Good Source** ■ A serving of the food contains at least 4 g of dietary fibre. **Excellent Source** ■ A serving of the food contains at least 7 g of dietary fibre.	**Source** ■ 2 g or more of dietary fibre per reference amount ^†^ and serving size. **High Source** ■ 4 g or more of dietary fibre per reference amount ^†^ and serving size. **Very High Source** ■ 6 g or more of dietary fibre per reference amount ^†^ and serving size.	**Source** ■ 3 g of dietary fibre per 100 g or at least 1.5 g of fibre per 100 kcal **High Source** ■ 6 g dietary of fibre per 100 g or at least 3 g of fibre per 100 kcal	**Good Source** ■ ≥10% to ≤19.9% of the DRV * for dietary fibre per RACC ^§^ of food **High Source** ■ ≥20% of the DRV * for dietary fibre per RACC ^§^ of food

Abbreviations: DRV: daily reference value; RACC, reference amount customarily consumed. ^†^ Canada: A reference amount is a regulated serving size that is typically consumed in a single meal event [26]. * US DRV for dietary fiber: Adults and children ≥4 years, 28 g/day; children 1–3 years, 14 g/day; pregnant and lactating women, 28 g/day ^§^ US: An RACC is a regulated serving size consumed in a single meal event [27].

**Table 2:** Definitions of fibre from Codex and regulatory agencies in Australia and New Zealand, Canada, Europe, and the US.

Jurisdiction	Definition of Dietary Fibre
Codex Alimentarius [31]	■ Carbohydrate polymers with ≥10 or more monomeric units (DP * ≥ 10), which are not hydrolyzed by the endogenous enzymes in the small intestine of humans and belong to the following categories: Edible carbohydrate polymers naturally occurring in the food as consumed; Carbohydrate polymers, which have been obtained from food raw material by physical, enzymatic, or chemical means and which have been shown to have a physiological effect of benefit to health as demonstrated by generally accepted scientific evidence to competent authorities; Synthetic carbohydrate polymers which have been shown to have a physiological effect of benefit to health as demonstrated by generally accepted scientific evidence to competent authorities.
Australia and New Zealand [20]	■ Dietary fibre means the fraction of the edible parts of plants or their extracts, or synthetic analogues, that are resistant to digestion and absorption in the small intestine, usually with complete or partial fermentation in the large intestine. ■ Dietary fibre includes polysaccharides, oligosaccharides (DP * > 2), and lignins, and promotes one or more of the following beneficial physiological effects: Laxation; Reduction in blood cholesterol; Modulation of blood glucose.
Canada [30]	■ Carbohydrates with a DP * ≥3 that naturally occur in foods of plant origin and that are not digested and absorbed by the small intestine; and ■ Accepted novel dietary fibres: Novel dietary fibres are ingredients manufactured to be sources of dietary fibre and consist of carbohydrates with a DP * of 3 or more that are not digested and absorbed by the small intestine. They are synthetically produced or are obtained from natural sources which have no history of safe use as dietary fibre or which have been processed so as to modify the properties of the fibre contained therein. Accepted novel dietary fibres have at least one physiological effect demonstrated by generally accepted scientific evidence: ▪ Improves laxation or regularity by increasing stool bulk; ▪ Reduces blood total and/or LDL-cholesterol levels; ▪ Reduces postprandial blood glucose and/or insulin levels, or increases sensitivity to insulin; ▪ Provides energy-yielding metabolites through colonic fermentation. Other physiological benefits of novel dietary fibres could be accepted.
Europe [28,29]	■ “Fibre” means carbohydrate polymers with 3 or more monomeric units, which are neither digested nor absorbed in the human small intestine and belong to the following categories: Edible carbohydrate polymers naturally occurring in the food as consumed; Edible carbohydrate polymers which have been obtained from food raw material by physical, enzymatic, or chemical means and which have a beneficial physiological effect demonstrated by generally accepted scientific evidence; Edible synthetic carbohydrate polymers which have a beneficial physiological effect demonstrated by generally accepted scientific evidence. ■ Accepted physiological benefits are not defined in Regulation 1169/2011. However, repealed Directive 90/496/EEC (replaced by regulation 1169/2011) indicated that that physiological benefits of dietary fibre include: Decrease intestinal transit time; Increase stool bulk; Fermentable by colonic microflora; Reduce blood total cholesterol, reduce blood LDL-cholesterol levels; Reduce postprandial blood glucose, or reduce blood insulin levels.
United States [32]	■ Dietary fibre is defined as non-digestible soluble and insoluble carbohydrates (DP * of ≥3 monomeric units), and lignin that are intrinsic and intact in plants; ■ Isolated or synthetic non-digestible carbohydrates (DP * of ≥3 monomeric units) determined by the FDA to have physiological effects that are beneficial to human health. Examples include: Attenuation of blood glucose and/or insulin levels; Reductions in fasting blood total and LDL-cholesterol levels; Improved laxation; Increased intestinal absorption of minerals; Reduced energy intake from food consumption.

Abbreviations: DP, degree of polymerization; FDA, US Food and Drug Administration; LDL, low-density lipoprotein; * DP refers to the number of monomeric units of the carbohydrate molecule.

**Table 3:** Summary of function-type health claims supported by dietary fibre in Australia and New Zealand, Canada, and Europe ^§^.

Region	Fibre Type	Claim	Claim Type	Criteria
Australia and New Zealand	Dietary Fibre [23]	Contributes to regular laxation	General level health claim *	■ Food meets the general conditions for making a nutrient content claim.
	Beta-glucan [23]	Reduces dietary and biliary cholesterol absorption	General level health claim *	■ One or more of the following oat or barley foods: ■ Oat bran; or ■ Whole grain oats; or ■ Whole grain barley; and ■ At least 1 g per serving beta-glucan from the abovementioned foods; and ■ Indicate that 3 g/day beta glucan is required; and ■ The food meets the nutritional criteria of the NPSC [23,33].
Canada	Psyllium fibre [34]	Increased laxation	Function claim	■ Food contains ≥3.5 g/serving psyllium fibre; or ■ If the food contains <3.5 g/serving psyllium fibre, the claim must indicate 3.5 g/day psyllium fibre promotes laxation or regularity.
	Wheat bran fibre [34]	Increased laxation	Function claim	■ Food contains ≥7 g/serving course wheat bran fibre; or ■ If the food contains <7 g/serving course wheat bran fibre, the claim must indicate 7 g/day course wheat bran fibre promotes laxation or regularity
	Polysaccharide complex (glucomannan, xanthan gum, sodium alginate) [35]	Lowers postprandial glycemic response	Function claim	■ Food contains ≥5 g per serving of stated size and reference amount ^†^ of polysaccharide complex; and ■ Food contains <15 g total sugars per serving of stated size and reference amount ^†^; or ■ Food contains <15 g total sugars per serving of stated size, if the food is a prepackaged meal, supplement, or meal replacement.
Europe	Barley grain fibre [36]	Increased laxation (increased fecal bulk)	Function health claim	■ Food contains sufficient barley grain fibre to qualify for a “high in fibre claim” (see Table 1)
	Rye fibre [36]	Normal bowel function	Function health claim	■ Food contains sufficient rye fibre to qualify for a “high in fibre claim” (see Table 1)
	Sugar beet fibre [37]	Increased laxation (increased fecal bulk)	Function health claim	■ Food contains sufficient sugar beet fibre to qualify for a “high in fibre claim” (see Table 1)
	Wheat bran fibre [36]	Increased laxation (increased fecal bulk)	Function health claim	■ Food contains sufficient wheat bran fibre to qualify for a “high in fibre claim” (see Table 1)
	Wheat bran fibre [36]	Laxation (decreased transit time)	Function health claim	■ Food contains sufficient wheat bran fibre to qualify for a “high in fibre claim” (see Table 1) ■ Information provided to the consumer that 10 g/day wheat bran fibre is required.
	Arabinoxylan produced from wheat endosperm [36]	Lowers postprandial glycemic response	Function health claim	■ Food contains at least 8 g of arabinoxylan fibre produced from wheat endosperm per 100 g of available carbohydrates in a quantified portion as part of the meal; and ■ Arabinoxylan fibre from wheat endosperm represents 60% arabinoxylan by weight; and ■ Information is provided to the consumer that the beneficial effect is obtained by consuming arabinoxylan-rich fibre as part of the meal.
	Beta-glucans from oats and barley [36]	Lowers postprandial glycemic response	Function health claim	■ Food which contains at least 4 g of beta-glucans from oats or barley for each 30 g of available carbohydrates in a quantified portion as part of the meal; and ■ Information is provided to the consumer that the beneficial effect is obtained by consuming the beta-glucans from oats or barley as part of the meal.
	Hydroxypropyl methylcellulose (HPMC) [36]	Lowers postprandial glycemic response	Function health claim	■ Food which contains at least 4 g of HPMC per quantified portion as part of the meal; and ■ Information is provided to the consumer that the beneficial effect is obtained by consuming HPMC as part of the meal; and ■ Warning of choking for people with swallowing difficulties; and ■ Advice on consuming with water to ensure HPMC reaches the stomach.
	Pectins [36]	Lowers postprandial glycemic response	Function health claim	■ 10 g of pectins per quantified portion; and ■ Information is provided to the consumer that the beneficial effect is obtained by consuming 10 g of pectins as part of the meal; and ■ Warning of choking for people with swallowing difficulties; and ■ Advice on consuming with water to ensure pectins reach the stomach.
	Resistant starch [36]	Lowers postprandial glycemic response	Function health claim	■ Food in which digestible starch has been replaced by resistant starch so that the final content of resistant starch is at least 14% of total starch.
	Beta-glucans [36]	Maintains normal blood cholesterol levels	Function health claim	■ The claim may be used only for food which contains at least 1 g of beta-glucans from oats, oat bran, barley, barley bran, or from mixtures of these sources per quantified portion; and ■ Information is provided to the consumer that the beneficial effect is obtained with a daily intake of 3 g of beta-glucans from oats, oat bran, barley, barley bran, or from mixtures of these beta-glucans.
	Glucomannan (Konjac mannan) [36]	Maintains normal blood cholesterol levels	Function health claim	■ Food provides at least 4 g/day of glucomannan; and ■ The claim indicates that the benefit is obtained with 4 g/day of glucomannan; ■ Warning of choking for people with swallowing difficulties; and ■ Advice on consuming with water to ensure glucomannan reaches the stomach.
	Guar Gum [36]	Maintains normal cholesterol levels	Function health claim	■ Food provides at least 10 g/day of guar gum; and ■ The claim indicates that the benefit is obtained with 10 g/day of guar gum; and ■ Warning of choking for people with swallowing difficulties; and ■ Advice on consuming with water to ensure guar gum reaches the stomach.
	Hydroxypropyl methylcellulose (HPMC) [36]	Maintains normal blood cholesterol levels	Function health claim	■ Food provides at least 5 g/day of HPMC; and ■ The claim indicates that the benefit is obtained with 5 g/day of HPMC; and ■ Warning of choking for people with swallowing difficulties; and ■ Advice on consuming with water to ensure HMPC reaches the stomach.
	Pectins [36]	Maintains normal blood cholesterol levels	Function health claim	■ Food provides at least 6 g/day of pectins; and ■ The claim indicates that the benefit is obtained with 6 g/day of pectins; and ■ Warning of choking for people with swallowing difficulties; and ■ Advice on consuming with water to ensure that pectins reach the stomach.
	Glucomannan (Konjac mannan) [36]	Contributes to weight loss in the context of an energy restricted diet	Function health claim	■ Food provides 1 g glucomannan per quantified portion; and ■ Information is provided to the consumer that the beneficial effect is obtained with 3 g/day glucomannan in 3 doses of 1 g each that is consumed with 1–2 glasses of water before meals in the context of an energy restricted diet.

Abbreviations: HPMC, hydroxypropyl methylcellulose; NPSC, Nutrient Profiling Scoring Criterion; RACC, reference amount customarily consumed. * Australia and New Zealand: A general level health claim refers to a claim that is not considered a high-level health claim. A high-level health claim refers to a serious disease or biomarker for a serious disease. A serious disease is a disease, disorder, or condition that is generally diagnosed, treated, or managed in consultation with or with supervision by a health care professional [38]. ^†^ Canada: A reference amount is a regulated serving size that is typically consumed in a single meal event [26]. ^§^ Structure/function health claims in the US for conventional foods do not require pre-approval and the US code of the federal registrar does not provide a list of corresponding claims [39].

**Table 4:** Summary of therapeutic and disease reduction claims supported by dietary fibre in Australia and New Zealand, Canada, Europe, and the US.

Region	Fibre Type	Claim	Claim Type	Criteria
Australia and New Zealand	Beta-glucan [23]	Reduces blood cholesterol	High-level health claim *	■ One or more of the following oat or barley foods: ■ Oat bran; or ■ Whole grain oats; or ■ Whole grain barley; and ■ At least 1 g per serving beta-glucan from the abovementioned foods; and ■ Claim is in the context of a diet low in saturated fatty acids; and ■ Indication that 3 g/day beta glucan is required; and ■ The food meets the nutritional criteria of the NPSC [23,33].
Canada	Barley beta-glucan [43]	Reduces cholesterol levels	Therapeutic claim	■ Food contains at least 1.0 g barley beta-glucan per reference amount ^†^ and per serving of stated size; and ■ Claim must indicate that 3 g/day beta-glucan from barley fibre lowers cholesterol levels; and ■ Food must meet specific nutritional requirements.
	Oat beta-glucan [42]	Reduces cholesterol levels	Therapeutic claim	■ Food contains at least 0.75 g oat beta-glucan per reference amount ^†^ and per serving of stated size; and ■ Claim must indicate 3 g/day beta-glucan from oat fibre lowers cholesterol levels; and ■ Food must meet specific nutritional requirements.
	Polysaccharide complex (glucomannan, xanthan gum, sodium alginate) [46]	Reduces cholesterol levels	Therapeutic claim	■ Food contains at least 3.3 g/of polysaccharide complex per reference amount ^†^ and per serving of stated size; and ■ Claim must indicate 10 g/day polysaccharide complex lowers cholesterol levels; and ■ Food must meet specific nutritional requirements.
	Psyllium [45]	Reduces cholesterol levels	Therapeutic claim	■ Food contains at least 1.75 g psyllium soluble fibre per reference amount ^†^ and per serving of stated size; and ■ Claim must indicate 7 g/day psyllium fibre lowers cholesterol levels; and ■ Food must meet specific nutritional requirements.
Europe	Barley beta-glucans [41]	Reduces cholesterol levels	Reduced disease risk factor health claim	■ The claim can be used for foods which provide at least 1 g of barley beta-glucan per quantified portion; and ■ Information is provided to the consumer that the beneficial effect is obtained with 3 g/day of barley beta-glucan.
	Oat beta-glucan [40]	Reduces cholesterol levels	Reduced disease risk factor health claim	■ The claim can be used for foods which provide at least 1 g of oat beta-glucan per quantified portion; and ■ Information is provided to the consumer that the beneficial effect is obtained with 3 g/day of oat beta-glucan.
United States	Barley beta-glucan [44]	May reduce risk of coronary heart disease	Authorized health claim	■ Food contains at least 0.75 g beta glucan soluble fibre from barley per RACC ^§^; and ■ Claim must indicate 3 g/day beta-glucan from barley fibre lowers cholesterol levels; and ■ Food must meet specific nutritional requirements.
	Oat beta-glucan [44]	May reduce risk of coronary heart disease	Authorized health claim	■ Food contains at least 0.75 g beta-glucan soluble fibre from oat per RACC ^§^; and ■ Claim must indicate 3 g/day beta-glucan from oat fibre lowers cholesterol levels; ■ Food must meet specific nutritional requirements.
	Psyllium husk [44]	May reduce risk of coronary heart disease	Authorized health claim	■ Food contains at least 1.7 g psyllium soluble fibre per RACC ^§^; and ■ Claim must indicate 7 g/day soluble psyllium fibre lowers cholesterol levels; and ■ Food must meet specific nutritional requirements.
	Fruit, vegetables, and grain products that contain soluble fibre [47]	May reduce risk of coronary heart disease	Authorized health claim	■ Food contains at least 0.6 g soluble fibre (without fortification) per RACC ^§^; and ■ Content of soluble fibre is listed in the nutrition information panel; and ■ Food must meet specific nutritional requirements.
	Fiber-containing grain products, fruits, and vegetables and cancer [48]	May reduce the risk of some types of cancers	Authorized health claim	■ Food meets the nutrient content requirements to be considered a “good source of fibre” (without fortification) (Table 1); and ■ Food must meet specific nutritional requirements.

Abbreviations: NPSC, nutrient profiling scoring criterion; RACC, reference amount customarily consumed. * Australia and New Zealand: A high-level health claim refers to a serious disease or biomarker for a serious disease. A serious disease is a disease, disorder, or condition that is generally diagnosed, treated, or managed in consultation with or with supervision by a health care professional [38]. ^†^ Canada: A reference amount is a regulated serving size that is typically consumed in a single meal event [21]. ^§^ US: RACC is a regulated serving size consumed at a single meal event [27].
